# Do Low Preoperative Vitamin D Levels Reduce the Accuracy of Quick Parathyroid Hormone in Predicting Postthyroidectomy Hypocalcemia?

**DOI:** 10.1245/s10434-012-2666-y

**Published:** 2012-09-12

**Authors:** Brian Hung-Hin Lang, Kai Pun Wong, Benjamin J. Cowling, Yuen Ki Fong, Desmond Kwan-Kit Chan, Grace Kin-Yee Hung

**Affiliations:** 1 Division of Endocrine Surgery, Department of Surgery, University of Hong Kong, Queen Mary Hospital, Pokfulam, Hong Kong SAR, China; 2 School of Public Health, The University of Hong Kong, Hong Kong SAR, China

## Abstract

**Background:**

Although some studies have suggested that low preoperative 25-hydroxyvitamin D (25-OHD) levels may increase the risk of hypocalcemia and decrease the accuracy of single quick parathyroid hormone in predicting hypocalcemia after total thyroidectomy, the literature remains scarce and inconsistent. Our study aimed to address these issues.

**Methods:**

Of the 281 consecutive patients who underwent a total/completion total thyroidectomy, 244 (86.8 %) did not require any oral calcium and/or calcitriol supplements (group 1), while 37 (13.2 %) did (group 2) at hospital discharge. 25-OHD level was checked 1 day before surgery, and postoperative quick parathyroid hormone (PTH) was checked at skin closure (PTH-SC). Postoperative serum calcium was checked regularly. Hypocalcemia was defined by the presence of symptoms or adjusted calcium of <1.90 mmol/L. Significant factors for hypocalcemia were determined by univariate and multivariate analyses. The accuracy of PTH-SC in predicting hypocalcemia was measured by area under a receiver operating characteristic curve (AUC), and the AUC of PTH-SC was compared between patients with preoperative 25-OHD <15 and ≥15 ng/mL via bootstrapping.

**Results:**

Preoperative 25-OHD level was not significantly different between groups 1 and 2 (13.1 vs. 12.5 ng/mL, *p* = 0.175). After adjusting for other significant factors, PTH-SC (odds ratio 2.49, 95 % confidence interval 1.52–4.07, *p* < 0.001) and parathyroid autotransplantation (odds ratio 3.23, 95 % confidence interval 1.22–8.60, *p* = 0.019) were the two independent factors for hypocalcemia. The AUC of PTH-SC was similar between those with 25-OHD <15 and ≥15 ng/mL (0.880 vs. 0.850, *p* = 0.61)

**Conclusions:**

Low 25-OHD was not a significant factor for hypocalcemia and did not lower the accuracy of quick PTH in predicting postthyroidectomy hypocalcemia.

Postoperative hypoparathyroidism leading to hypocalcemia is the one of the most frequent morbidities after total thyroidectomy with a reported incidence ranging 3–40 %.^[^ 1 ^,^ 2 ^]^ Because potentially life-threatening hypocalcemia does not usually develop until 24–48 h after surgery, hypoparathyroidism is one of the major reasons for delaying hospital discharge and dissuading surgeons from ambulatory thyroid surgery.^[^ 3 ^,^ 4 ^]^ To safely manage postoperative hypoparathyroidism/hypocalcemia and potentially shorten hospital stay, previous authors have suggested the use of single postoperative quick parathyroid hormone (qPTH) testing for predicting of postthyroidectomy hypocalcemia.^[^ 3 ^–^ 7 ^]^ Benefits of single qPTH over other strategies such as serial calcium (Ca) monitoring or serial percentage drop in postoperative parathyroid hormone (PTH) are the reduced need for multiple blood taking and shortened hospital stay.^[^ 6 ^]^ Similar predictive accuracies have been reported when it is taken at skin closure or up to several hours after surgery.^[^ 8 ^,^ 9 ^]^ We preferred the qPTH level taken at skin closure (PTH-SC) because it is convenient and does not inflict additional discomfort on patients.^[^ 6 ^]^

However, it remains unclear whether preoperative vitamin D level [as measured by 25-hydroxyvitamin D (25-OHD)] affects the accuracy of single qPTH in predicting postoperative hypocalcemia. The current literature suggests that it may be because some studies have reported that preoperative 25-OHD is a significant factor for postoperative hypocalcemia.^[^ 10 ^–^ 12 ^]^ A recent study also showed that the accuracy [when measured by the area under a receiver operating characteristic curve (AUC)] of qPTH in predicting hypocalcemia might be lower in patients with low 25-OHD compared to patients with normal 25-OHD.^[^ 13 ^]^ Therefore, we hypothesized that 25-OHD may be a significant factor for hypocalcemia, and a low 25-OHD level may affect the accuracy of qPTH in predicting postthyroidectomy hypocalcemia. However, because the evidence remains scarce and somewhat inconsistent, our study aimed to find out whether the 25-OHD level was a significant factor for hypocalcemia and to compare the predictive accuracy of qPTH between patients with different levels of 25-OHD.

## Methods and Patients

From March 2011 to April 2012, all consecutive patients who underwent either a total or completion total thyroidectomy were analyzed. All operations were performed by the same surgical team. The present study protocol was approved by the local institutional review board. The preoperative 25-OHD was checked 1 day before surgery, whereas the PTH-SC level was taken at skin closure (i.e., approximately 5–10 min after excision of the thyroid gland) while the patient was still anesthetized. Another qPTH level was taken on the next morning (approximately 18–24 h after operation) on postoperative day 1 (PTH-D1). Serum Ca and phosphate were checked preoperatively, within an hour after operation, on the next morning and every 8–10 h until Ca stabilized. There were 299 consecutive patients who underwent total or completion total thyroidectomy over this period. Those with concomitant selective central and/or lateral neck dissection (*n* = 10) and incomplete 25-OHD or qPTH values (*n* = 8) were excluded. Therefore, there were 281 patients (93.9 %) eligible for analysis. Eighty-one patients were previously included in another study, but because the 25-OHD data were not reported, they were included in the present study.^[^ 6 ^]^

Clinically significant hypocalcemia (hypocalcemia) was defined either by the presence of hypocalcemic symptoms or when the postoperative serum Ca dropped <1.90 mmol/L (normal range 2.11–2.55 mmol/L). Those with hypocalcemia were provided 500–1,500 mg oral Ca tablets initially, and additional calcitriol was considered, up to 0.50 μg twice daily, if Ca tablets alone failed to maintain normocalcemia. The person responsible for prescribing oral Ca with or without calcitriol supplements was unaware of the 25-OHD, PTH-SC, or PTH-D1 results. The primary end point of the study was hypocalcemia needing Ca with or without calcitriol supplements at hospital discharge. At hospital discharge, 244 patients (86.8 %) did not require any oral Ca with or without calcitriol (group 1), and 37 (13.2 %) did (group 2). Potential factors for hypocalcemia were determined by comparing patient characteristics and perioperative biochemical variables between groups 1 and 2.

Surgical techniques, postoperative care, and follow-up protocol had been previously described.^[^ 2 ^,^ 14 ^]^ Thyroidectomy was performed in a standardized manner with the patient under general anesthesia. The strap muscles were separated in the midline and retracted laterally. Both recurrent laryngeal nerves and parathyroid glands were routinely identified and preserved. Any devascularized parathyroid glands were immediately minced and autoimplanted to the ipsilateral sternocleidomastoid muscle. All postoperative patients were seen within 1 week and were asked specifically about hypocalcemic symptoms after hospital discharge. All data including patient biochemistry and supplements at discharge were prospectively recorded.

### Laboratory Methods

Serum albumin–adjusted Ca and phosphate levels were measured in the hospital laboratory by standard methods with the Modular Analytic system (Roche Diagnostics, Indianapolis, IN). qPTH level was measured by Access 2 immunoassay system (Beckman Coulter, Brea, CA), and the inter- and intra-assay coefficients of variation (CVs) were 5.8 and 4.5 %, respectively. The normal range for serum qPTH level was 1.2–5.7 pmol/L (to convert pmol/L to ng/L, divide by 0.106). Serum 25-OHD was measured with the electrochemiluminescence immunoassay (Elecsys Vitamin D Total assay), and the inter- and intra-assay CVs were 6.2 and 4.8 %, respectively. The measuring range was 3.00–70.0 ng/mL (to convert ng/mL to nmol/L, multiply by 2.496).

### Statistical Analysis

For comparison of dichotomous variables between two groups, chi-square tests and Fisher’s exact tests were used. The Mann-Whitney *U* test was used for comparison of continuous variables. Any preoperative and perioperative biochemical variables that were significant in the univariate analysis were entered into multivariate analysis to determine independent factors. To improve clinical utility of significant continuous variables, Youden’s index was used to calculate the best cutoff value for predicting hypocalcemia.^[^ 15 ^]^ The AUC was used to measure the predictive accuracy. AUC values closer to 1 meant better predictability, whereas values closer to 0.5 meant poorer predictability. A bootstrap approach with 1,000 resamples was used to estimate 95 % confidence intervals (CIs) for AUC and to compare two AUCs. All statistical analyses were conducted by SPSS software, version 18.0 (SPSS, Chicago, IL), and R software, version 2.14.0 (R Foundation for Statistical Computing, Vienna, Austria). *p* < 0.05 was considered statistically significant.

## Results

In this cohort, the median age was 53.2 (22.9–87.4) years, and the male:female ratio was 1:7.3. Two hundred sixty-one patients (92.9 %) underwent a total thyroidectomy, and 20 (7.1 %) underwent a completion total thyroidectomy. Of the 37 patients (13.2 %) requiring supplements at discharge (group 2), 14 (5.0 %) required Ca tablets alone, and 23 (8.2 %) required both Ca and calcitriol supplements. At follow-up, none experienced unexpected hypocalcemic symptoms or required readmission. The median 25-OHD level was 12.9 (3.0–33.1) ng/mL, with 83 patients (29.5 %) having 25-OHD <10 ng/mL.

Table 1 lists patient baseline characteristics, operative findings, and perioperative biochemistry between group 1 and group 2. Age at operation, sex ratio, surgical indications, preoperative thyroid-stimulating hormone level, blood loss, weight of excised gland, and size of dominant nodule were similar between the two groups. Group 2 had a significantly higher incidence of concomitant thyroiditis (27.0 vs. 11.5 %, *p* = 0.005) and longer duration of operation (68 vs. 53 min, *p* = 0.002) than group 1. The latter probably reflected the difficulty of the operation. Group 2 also had significantly higher proportion of parathyroid gland autotransplantation (64.9 vs. 20.5 %, *p* = 0.019). Interestingly, both preoperative adjusted Ca and 25-OHD levels were similar in the two groups. As expected, both PTH-SC and PTH-D1 were significantly higher in group 1 than group 2 (3.5 vs. 0.7 pmol/L, *p* < 0.001 and 2.5 vs. 0.3 pmol/L, *p* < 0.001, respectively). PTH-SC level significantly correlated with PTH-D1 (ρ = 0.634, *p* < 0.001).

**Table 1:** Comparison of baseline characteristics and perioperative biochemistry between those with normocalcemia (group 1) and with hypocalcemia (group 2) after surgery

Variable	Group 1 (*n* = 244)	Group 2 (*n* = 37)	*p*
Age at operation, years	54.0 (26.2–87.4)	48.3 (22.9–76.5)	0.246
Gender, M:F	32:212	2:35	0.278
Surgical indication/final pathology			0.323
Graves disease/toxic MNG	10 (4.2)	6 (16.2)	
Benign pathology	210 (86.1)	29 (78.4)	
Malignancy	24 (9.8)	2 (5.4)	
Preoperative TSH level, mIU/L	1.0 (0.03–5.1)	0.96 (0.03–4.9)	0.342
Concomitant autoimmune thyroiditis	28 (11.5)	10 (27.0)	**0.005**
Duration of operation, min	53 (39–185)	68 (46–140)	**0.002**
Blood loss, mL	10 (5–110)	10 (10–60)	0.116
Weight of excised gland, g	33.7 (6–162)	39.0 (16–74)	0.251
Size of dominant nodule, cm	2.0 (0.4–6.5)	2.75 (0.5–3.5)	0.677
No. of parathyroid glands identified			0.107
0	14 (5.7)	0 (0.0)	
1	14 (5.7)	1 (2.7)	
2	59 (24.2)	11 (29.7)	
3	61 (25.0)	15 (40.5)	
4	96 (39.3)	11 (29.7)	
Parathyroid autotransplantation	50 (20.5)	24 (64.9)	**<0.001**
Preoperative adjusted calcium, mmol/L	2.28 (2.13–2.47)	2.29 (2.12–2.44)	0.981
Preoperative 25-OHD level, ng/mL	13.1 (3.1–33.1)	12.5 (3.0–23.9)	0.175
PTH-SC, pmol/L	3.5 (0.3–16.8)	0.7 (0.2–4.9)	**<0.001**
PTH-D1, pmol/L	2.5 (0.3–11.0)	0.3 (0.3–6.1)	**<0.001**

Bold values indicate *p* < 0.05

*MNG* multinodular goiter, *TSH* thyroid-stimulating hormone, *25-OHD* 25-hydroxyvitamin D, *PTH-SC* postoperative quick intact parathyroid hormone at skin closure, *PTH-D1* quick intact parathyroid hormone level on postoperative day 1

Data are presented as *n* (%) or median (range)

Table 2 shows a multivariable analysis of risk factors for postoperative hypocalcemia. After adjusting for PTH-D1 level, duration of operation and concomitant autoimmune thyroiditis, the PTH-SC level [β coefficient = 0.912, odds ratio (OR) 2.488 (95 % CI 1.520–4.065), *p* < 0.001] and parathyroid autotransplantation (β coefficient = 1.173, OR 3.231, 95 % CI 1.215–8.597, *p* = 0.019) were the two independent factors for hypocalcemia. As PTH-SC significantly correlated with PTH-D1, the multivariate analysis was repeated with PTH-D1 removed. However, PTH-SC and parathyroid autotransplantation remained independent factors for hypocalcemia.

**Table 2:** Multivariable analysis of risk factors for postoperative hypocalcemia requiring calcium and/or vitamin D supplementation after surgery

Covariate	β coefficient	Odds ratio (95 % confidence interval)	*p*
PTH-SC, pmol/L	0.912	2.488 (1.520–4.065)	**<0.001**
PTH-D1, pmol/L	0.073	1.075 (0.687–1.684)	0.750
Duration of operation, min	0.002	1.002 (0.985–1.019)	0.810
Concomitant autoimmune thyroiditis	1.179	3.252 (0.837–12.627)	0.144
Parathyroid autotransplantation	1.173	3.231 (1.215–8.597)	**0.019**

Bold values indicate *p* < 0.05

*PTH-SC* postoperative quick intact parathyroid hormone at skin closure, *PTH-D1* quick intact parathyroid hormone level on postoperative day 1

Table 3 shows a comparison of test sensitivity, specificity, and predictability as measured by AUC between preoperative 25-OHD, PTH-SC, PTH-D1, and PTH-SC when 25-OHD <15 and ≥15 ng/mL. The best cutoff value in predicting hypocalcemia for PTH-SC was 1.5 pmol/L (Youden’s index 0.618, sensitivity 73.0 %, specificity 88.9 %) and for PTH-D1 was 0.9 pmol/L (Youden’s index 0.589, sensitivity 70.3 %, specificity 90.6 %). In terms of overall predictability (as measured by AUC), PTH-SC had a higher AUC value (0.866) than preoperative 25-OHD (AUC = 0.570) and PTH-D1 (AUC = 0.818). Because PTH-SC had the highest AUC, it was chosen for comparing accuracy between those with 25-OHD <15 and ≥15 ng/mL. The overall predictability (AUC) of PTH-SC was similar between those with 25-OHD <15 and ≥15 ng/mL (0.880 vs. 0.850, *p* = 0.61).

**Table 3:** Comparison of test sensitivity, specificity, and predictability

Level	Best cutoff value^a^	Sensitivity^b^	Specificity^c^	AUC (95 % CI)
Preoperative 25-OHD level, ng/mL	<17, ≥17	33/37 (89.2 %)	80/244 (32.8 %)	0.570 (0.476–0.644)
PTH-SC, pmol/L	<1.5, ≥1.5	27/37 (73.0 %)	217/244 (88.9 %)	0.866 (0.800–0.931)
PTH-D1, pmol/L	<0.9, ≥0.9	26/37 (70.3 %)	221/244 (90.6 %)	0.818 (0.084–0.280)
PTH-SC, pmol/L, when 25-OHD <15 ng/mL	<1.5, ≥1.5	17/23 (73.9 %)	133/144 (92.3 %)	0.880 (0.791–0.969)^d^
PTH-SC, pmol/L, when 25-OHD ≥15 ng/mL	<1.5, ≥1.5	10/14 (71.4 %)	84/100 (84.0 %)	0.850 (0.771–0.929)^d^

*AUC* area under the receiver operating characteristic curve, *CI* confidence interval, *25-OHD* 25-hydroxyvitamin D, *PTH-SC* postoperative quick intact parathyroid hormone at skin closure, *PTH-D1* quick intact parathyroid hormone level on postoperative day 1

^a^Determined by the ROC curve and Youden’s index^[^ 15 ^]^

^b^Test sensitivity = [truly positive/(truly positive + falsely negative)]

^c^Test specificity = [truly negative/(truly negative + falsely positive)]

^d^ *p* value for the difference between the 2 AUCs was 0.61

**Fig. 1:**
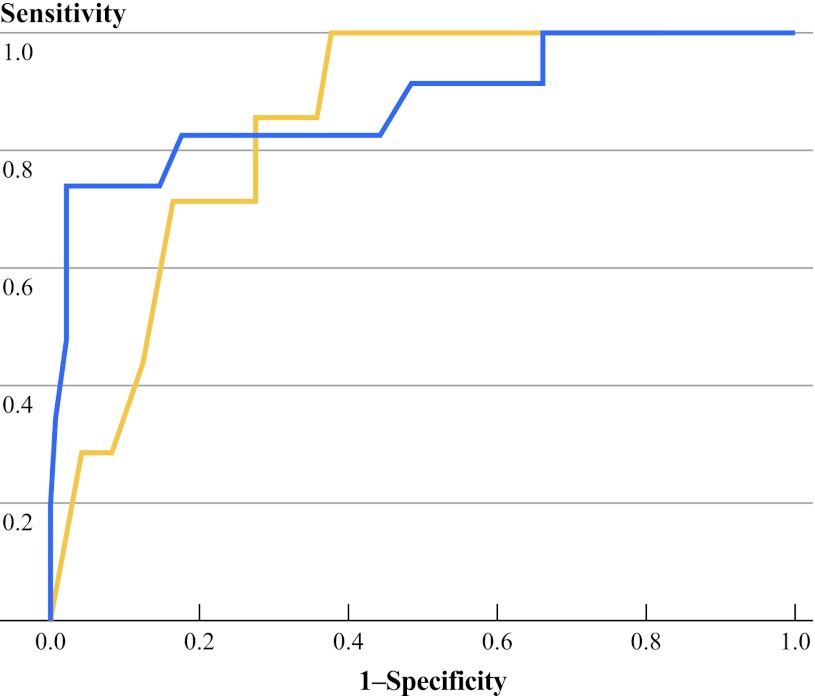
Areas under the receiver operating characteristic curve for quick parathyroid hormone at skin closure in the group with 25-hydroxyvitamin D (25-OHD) <15 ng/mL (*blue line*) and the group with 25-OHD ≥15 ng/mL (*orange line*)

Figure 1 shows the AUC of PTH-SC in the group with 25-OHD <15 ng/mL and the group with 25-OHD ≥15 ng/mL.

Table 4 shows the subgroup analysis of 25-OHD and PTH-SC levels between groups 1 and 2 after surgery. In the subgroup with PTH-SC <1.5 pmol/L, group 1 had significantly higher median 25-OHD (17.2 vs. 12.6 ng/mL, *p* = 0.025) and PTH-SC (1.2 vs. 0.6 pmol/L, *p* = 0.019) levels than those in group 2. However, when both factors were entered into multivariate analysis, PTH-SC (β coefficient = 1.919, OR 6.803, 95 % CI 1.570–29.412, *p* = 0.010) was the only independent factor for hypocalcemia. In the subgroup with PTH-SC ≥1.5 pmol/L, PTH-SC tended to be higher in group 1 but did not reach significance (*p* = 0.063). In group 2, the proportion requiring both Ca and calcitriol supplements (instead of Ca tablets only) was similar between PTH-SC <1.5 and ≥1.5 pmol/L (17 of 27, 63.0 %, vs. 6 of 10, 60.0 %, *p* = 1.000).

**Table 4:** Subgroup comparison of 25-OHD and PTH-SC between those with normocalcemia (group 1) and with hypocalcemia (group 2) after total thyroidectomy

Variable	Group 1 (*n* = 244)	Group 2 (*n* = 37)	Univariate *p* value	Multivariate *p* value^a^
PTH-SC <1.5 pmol/L (*n* = 54)				
25-OHD level, g/mL	17.2 (7.3–31.4)	12.6 (3.5–24.0)	**0.025**	0.081
PTH-SC level, pmol/L	1.2 (0.3–1.4)	0.6 (0.2–1.4)	**0.019**	**0.010**
PTH-SC ≥1.5 pmol/L (*n* = 227)				
25-OHD level, g/mL	12.7 (3.1–33.1)	12.3 (3.0–18.3)	0.215	–
PTH-SC level, pmol/L	3.9 (1.6–16.8)	2.9 (1.9–4.9)	0.063	–

Bold values indicate *p* < 0.05

*25-OHD* 25-hydroxyvitamin D, *PTH-SC* postoperative quick intact parathyroid hormone at skin closure

^a^Multivariate analysis was performed when both factors (25-OHD and PTH-SC) were significant (*p* < 0.05) in the univariate analysis

## Discussion

Because 25-OHD level plays an essential role in both Ca and PTH regulation, it has been hypothesized that perhaps preoperative 25-OHD level could be a significant factor affecting the risk of hypocalcemia and altering the accuracy of qPTH in predicting hypocalcemia after total thyroidectomy.^[^ 16 ^]^ Our study aimed at addressing these two related issues by evaluating whether preoperative 25-OHD was a significant factor in postthyroidectomy hypocalcemia and examining whether preoperative 25-OHD level affected the accuracy of single qPTH in predicting hypocalcemia. For the first issue, several studies found preoperative 25-OHD could be a significant factor of hypocalcemia.^[^ 10 ^–^ 12 ^,^ 17 ^]^ Erbil et al. reported the results of 130 patients with nontoxic multinodular goiter undergoing total thyroidectomy and found preoperative 25-OHD level to be an independent factor for postoperative hypocalcemia (OR 558.5, 95 % CI 27.6–11, 291.9). They also reported that 25-OHD was a more significant factor than 12 h PTH and age for hypocalcemia.^[^ 10 ^]^ A recent study similarly reported that the preoperative 25-OHD level was a significant factor for hypocalcemia, with patients with 25-OHD ≤14 ng/mL having significantly greater risk of hypocalcemia than 25-OHD >14 ng/mL.^[^ 12 ^]^ This appeared independent of the postoperative PTH level.^[^ 12 ^]^ As a result, some authors suggested routine Ca or vitamin D supplementation for patients with preoperative 25-OHD <15 ng/mL to avoid postoperative hypocalcemia.^[^ 11 ^,^ 18 ^]^ However, the association between low 25-OHD and hypocalcemia has not been consistently demonstrated with some studies showing no significant association.^[^ 3 ^,^ 19 ^]^ Our study showed that there was no significant association between 25-OHD and hypocalcemia. When patient characteristics and biochemistry were compared between groups 1 and 2, 25-OHD level was not significantly different (13.1 vs. 12.5 ng/mL, *p* = 0.175). Instead, the PTH-SC (OR 2.49) and parathyroid autotransplantation (OR 3.23) were the two most significant factors for hypocalcemia. Other studies also found similar findings.^[^ 20 ^,^ 21 ^]^ The reasons why parathyroid autotransplantation might be significant are 2-fold. First, there might have been a selection bias, with patients perceived of having higher risk of hypocalcemia more likely undergoing parathyroid autotransplantation. Second, although parathyroid autotransplantation may reduce permanent hypocalcemia in the long term, it tends to increase temporary hypocalcemia.^[^ 22 ^]^

Given that we did not find a significant association between 25-OHD and hypocalcemia, we proceeded with a post hoc subgroup analysis (Table 4) looking at the two different patient subgroups of PTH-SC, <1.5 and ≥1.5 pmol/L. We postulated that perhaps patients with low qPTH (i.e., PTH < 1.5 pmol/L) might have been more susceptible to hypocalcemia when the 25-OHD was low than when 25-OHD was normal. Although we did find group 2 had significantly lower 25-OHD level than group 1, when both 25-OHD and PTH-SC were entered into a multivariate analysis, only PTH-SC was a statistically significant factor for hypocalcemia. Therefore, a low 25-OHD may only have a minor or weak effect on hypocalcemia even when PTH-SC is <1.5 pmol/L, and conversely, a normal 25-OHD level may not be able to compensate for the low qPTH level in preventing hypocalcemia. It was also interesting that when only group 2 patients were considered, the proportion needing both Ca with calcitriol supplementation was similar between PTH-SC <1.5 and ≥1.5 pmol/L (63.0 vs. 60.0 %, *p* = 1.000). This was despite the fact that the former group had significantly lower PTH-SC level than the latter group (0.6 vs. 2.9 pmol/L, *p* < 0.001). One explanation for this discrepancy may be that despite a detectable PTH by the assay, the amount of PTH available in the serum to maintain normocalcemia remained low.^[^ 23 ^]^ Furthermore, there might have been other factors, including parathyroid autotransplantation, that could influence the risk of hypocalcemia; in addition, PTH-SC was not a test with 100 % accuracy.

In our second analysis, we aimed to find out whether low preoperative 25-OHD level affected the accuracy of single qPTH in predicting hypocalcemia by comparing the AUC of PTH-SC when 25-OHD <15 ng/mL with the AUC of PTH-SC when 25-OHD ≥15 ng/mL. However, unlike a previous study reporting that the AUC of qPTH was lower in 25-OHD <10 ng/mL than in ≥10 ng/mL (0.68 vs. 0.93), we found that the two groups had similar accuracy by AUC (0.88 vs. 0.85, *p* = 0.61).^[^ 13 ^]^ This was despite our study having a significant larger cohort. In other words, PTH-SC had similar accuracy in predicting postthyroidectomy hypocalcemia regardless of whether the preoperative 25-OHD was <15 or ≥15 ng/mL. However, because the number of studies focusing on this issue is small, our finding requires further confirmation by future prospective studies.

Our data support the view that routine testing of serum 25-OHD level before total thyroidectomy is unnecessary because it neither predicts nor affects the risk of hypocalcemia. Regarding whether routine preoperative vitamin D supplementation would be beneficial in patients who undergo total thyroidectomy, this was not addressed and will require further evaluation. One of the shortcomings of our study was that because a sample calculation was not performed, our findings might have been purely the result of the underpowering of the study. However, this study was one of the largest studies of its kind with all data collected prospectively.

In conclusion, PTH-SC and parathyroid autotransplantation were the two independent factors or predictors for hypocalcemia after thyroidectomy, whereas preoperative 25-OHD level was not. Preoperative 25-OHD level was also not a significant factor for hypocalcemia in patients with PTH-SC <1.5 pmol/L. The accuracy of PTH-SC in predicting hypocalcemia appeared similar between patients with preoperative 25-OHD <15 and ≥15 ng/mL.
